# Effect of a Mediterranean Diet-Based Nutritional Intervention on the Risk of Developing Gestational Diabetes Mellitus and Other Maternal-Fetal Adverse Events in Hispanic Women Residents in Spain

**DOI:** 10.3390/nu12113505

**Published:** 2020-11-14

**Authors:** Verónica Melero, Nuria García de la Torre, Carla Assaf-Balut, Inés Jiménez, Laura del Valle, Alejandra Durán, Elena Bordiú, Johanna J. Valerio, Miguel A Herraiz, Nuria Izquierdo, Maria José Torrejón, Isabelle Runkle, Ana Barabash, Miguel A Rubio, Alfonso L Calle-Pascual

**Affiliations:** 1 Endocrinology and Nutrition Department, Hospital Clínico Universitario San Carlos and Instituto de Investigación Sanitaria del Hospital Clínico San Carlos (IdISSC), E 28040 Madrid, Spain; veronica.meleroalvarez10@gmail.com (V.M.); nurialobo@hotmail.com (N.G.d.l.T.); carlaassafbalut90@hotmail.co.uk (C.A.-B.); i.jimenez.varas@gmail.com (I.J.); lauradel_valle@hotmail.com (L.d.V.); aduranrh@hotmail.com (A.D.); elena.bordiu@salud.madrid.org (E.B.); valeriojohanna@gmail.com (J.J.V.); irunkledelavega@gmail.com (I.R.); ana.barabash@gmail.com (A.B.); marubioh@gmail.com (M.A.R.); 2 Centro de Investigación Biomédica en Red de Diabetes y Enfermedades Metabólicas Asociadas (CIBERDEM), E 28040 Madrid, Spain; 3 Medicina 2 Department, Facultad de Medicina, Universidad Complutense de Madrid, E 28040 Madrid, Spain; maherraizm@gmail.com (M.A.H.); nuriaizquierdo4@gmail.com (N.I.); 4 Gynecology and Obstetrics Department, Hospital Clínico Universitario San Carlos and Instituto de Investigación Sanitaria del Hospital Clínico San Carlos (IdISSC), E 28040 Madrid, Spain; 5 Clinical Laboratory Department, Hospital Clínico Universitario San Carlos and Instituto de Investigación Sanitaria del Hospital Clínico San Carlos (IdISSC), E 28040 Madrid, Spain; mjosetorrejon@gmail.com

**Keywords:** gestational diabetes mellitus, Hispanic ethnicity, Mediterranean diet, nutritional intervention, pregnancy

## Abstract

Gestational diabetes mellitus (GDM) is the most frequent morbidity found in pregnancy, and it increases the risk for several maternal-fetal complications. Hispanic women are considered at high risk. The St. Carlos GDM prevention study is a randomized controlled trial (RCT) conducted from 2016–2017. Normoglycemic women were randomized at 12–14 Gestation week (WG) to an intervention group (IG) receiving recommendations based on the MedDiet (supplemented with ExtraVirgin Olive Oil/pistachios), or to a control group (CG), recommended to limit fat intake. After RCT conclusion, IG recommendations were applied to a real-world group (RW) in routine clinical practice. The primary endpoint of the current study is an assessment of the GDM rate in Hispanic participants of the aforementioned studies: 132 RCT, 128 CT, 284 RW participants. The GDM rate was lower in IG: 19/128(14.8%), *p* = 0.021, and RW: 38/284(13.4%), *p* = 0.029) than in CG: 34/132(25.8%). Adjusted RR (95%CI) for GDM: 0.72 (0.50–0.97), *p* = 0.037 in IG and 0.77 (0.61–0.97), *p* = 0.008 in RW. Rates of urinary tract infections, emergency caesarean-sections and perineal trauma were also lower in IG and RW. Other adverse outcomes were lower in IG vs. CG. In conclusion, a MedDiet-based intervention reduces the rate of GDM and several adverse maternal-fetal outcomes in Hispanic women residing in Spain.

## 1. Introduction

Gestational diabetes mellitus (GDM) is the most frequently encountered health problem in pregnancy, and it is associated with an increased risk of suffering diverse adverse maternal-fetal outcomes [1,2,3,4]. Different nutritional interventions for the prevention of GDM have been recently reviewed. However, the results of published meta-analyses have been inconclusive [5,6,7,8]. Randomized controlled trials (RCTs) evaluating the effects of different types of nutritional intervention have found divergent effects on women’s prenatal risk [9,10,11,12,13].

The effects of the Mediterranean diet (MedDiet) during pregnancy have been recently reviewed [14,15]. In fact, our own group has demonstrated that this diet can reduce the occurrence of GDM both in RCT [10] as well as in routine clinical practice [16]. However, most of the participants were considered to be at a low risk for the development of GDM, with a normal pre-gestational weight, and with the majority being of Caucasian ethnicity. 

Hispanic women represent the largest ethnic minority in Madrid and have a higher birth rate than Caucasian women. The city of Madrid had 11,165 female residents who had emigrated from Latin America on January 1st, 2020 [17]. Hispanic women are considered to be at high risk for development of GDM. Indeed, Hispanic women living in the United States have the highest age-standardized relative increase in the diagnosis of GDM when compared with other ethnicities [18], and fetal macrosomia is more frequent. In fact, abnormal fetal growth, which varies with ethnicity, is associated with GDM, preconception obesity, and maternal weight gain during pregnancy [19,20]. It is therefore crucial to develop preventive strategies to reduce the incidence of GDM in Hispanic women. 

Health is influenced by a complex interplay of individual factors, including genetics, lifestyle, ethnicity, and social determinants such as immigrant status [21]. In fact, Hispanic women could have an increased risk of developing GDM [22] due to both genetic predisposition and lifestyle habits [23,24,25]. Hispanic women residing in United States have a larger waist circumference compared with women of other origins. They also have a higher probability for the development of diabetes and the metabolic syndrome (MetS) [25].

Our group published that women of Hispanic origin residing in Spain acquire a similar lifestyle to that of the native Spanish population when residing in Spain for over 3 years [26]. Whether a nutritional intervention based on MedDiet is effective in preventing GDM in this high-risk ethnic group residing in Spain remains to be elucidated.

The aim of this paper is to evaluate whether an early MedDiet-based nutritional intervention implemented in the first antenatal visit reduces the incidence of GDM and other maternal-fetal adverse events in Hispanic women who participated in both RCT [10] and the study based on the implementation of this research into real-world (RW) clinical practice [16].

## 2. Materials and Methods

### 2.1. Study Design

The St. Carlos GDM prevention study is a prospective, clinic-based, interventional RCT conducted from 2015 to 2016 at the Hospital Clínico San Carlos, Madrid, Spain. Normoglycemic women who attended their first gestational visit at 8–12 gestational weeks (GW) were invited to participate, and randomized by age, ethnicity, body mass index (BMI) and parity before the 14th GW to an intervention group (IG) or a control group (CG). 

After evaluating the results of this RCT, and based on the positive outcomes observed, the nutritional guidelines provided to the IG were adopted as standard nutritional management. From October 2016 on, every pregnant woman who attended the first gestational visit received nutritional recommendations in compliance with these guidelines, as routine clinical practice. To analyze the reproducibility of the results of the RCT and the potential application of the research into practice, another study was carried out over a one-year period. The first 1000 consecutive pregnant women who came to our center between November 2016 and throughout the year 2017 for follow-up were invited to participate in the study, based on the implementation of the RCT results in clinical practice. There were no distinctions regarding the number of visits with members of the attending team (obstetricians, dieticians, diabetes nurse educators, and endocrinologists) in this RW study when compared with the RCT. 

The RCT was approved by the Ethics Committee of Hospital Clínico San Carlos (CI 13/296-E), and it registered with the number ISRCTN84389045 (DOI 10.1186/ISRCTN84389045). The study based on the routine clinical practice was also approved (16/442-E), and it registered with the number ISRCTN13389832 (DOI 10.1186/ISRCTN13389832). Both studies were conducted according to the Helsinki Declaration. All women signed a letter of informed consent.

### 2.2. Participants 

A total of 600 women of Hispanic origin (142/CG, 143/IG and 315/RW) were included in the aforementioned RCT and extension studies, and they are the subject of the current study. All agreed to participate in the study and signed the informed consent. Women’s demographic and clinical characteristics at baseline can be seen in Table 1.

In total, 56 women were lost to the follow-up, and, therefore, 544 women were assessed (132/CG, 128/IG and 284/RW). The country of origin of the participants can be seen in Table S1 online.

### 2.3. Pregnancy Follow-Up 

At the inclusion visit (8–12 GW) women had to meet inclusion criteria (fasting blood glucose (FBG) <92 mg/dL; ≥18 years of age, and single pregnancy) and have no exclusion criteria (gestational age above 14 GW, allergy or intolerance to nuts or EVOO, or another medical contraindication). All women signed the consent form.

At visit 1 (12–14 GW), after performing their first prenatal ultrasound, the nutritional intervention was applied. In summary, patients included in the CG were advised to restrict fat intake, with the consumption of extra virgin olive oil (EVOO) limited to a maximum of 40 mL/day, and nuts <3 days per week as usually recommended. Women of IG and RW were advised to increase their consumption of EVOO (≥40 mL/day) and have a handful of pistachios (25–30 g) at least 3 days a week. 10 L of EVOO (about 1 L each 10 days) and 2 Kg of roasted pistachios (about 160 g a week) were provided at no cost to women in the IG at visits 1 and 2 in order to ensure the consumption of the minimum amount recommended throughout pregnancy. The pistachio was chosen as a dried fruit in the study since those used for the study were of Spanish production, and it has also been seen that the fact that the pistachio does not come peeled but with the shell stimulates the cerebral cephalic phase, which is why it is interesting compared to other nuts. These recommendations were also given to women belonging to RW, although they were not given a free supply of EVOO or pistachios. 

At visit 2 (24–28 GW) the nutritional intervention was strengthened by a meeting of approximately 2 h with a dietician. MedDiet nutritional advice was reinforced in the IG and RW groups, and participants told to increase their consumption of EVOO and nuts. CG participants were again told to limit fat intake. GDM screening was performed using the 75 g oral glucose tolerance test (OGTT), according to International Association of Diabetes and Pregnancy Study Groups (IADPSG) criteria [27]. If GDM was diagnosed, women were derived to the Diabetes and Pregnancy Unit to be treated with insulin (which was tapered weekly) or diet as appropriate, based on fasting and 1-h postprandial capillary blood glucose values, as previously reported [28].

At visit 3 (36–38 GW) and 4 (at delivery), adherence to nutritional recommendations, pregnancy progression and delivery, and neonatal outcomes were assessed.

**Figure 1:**
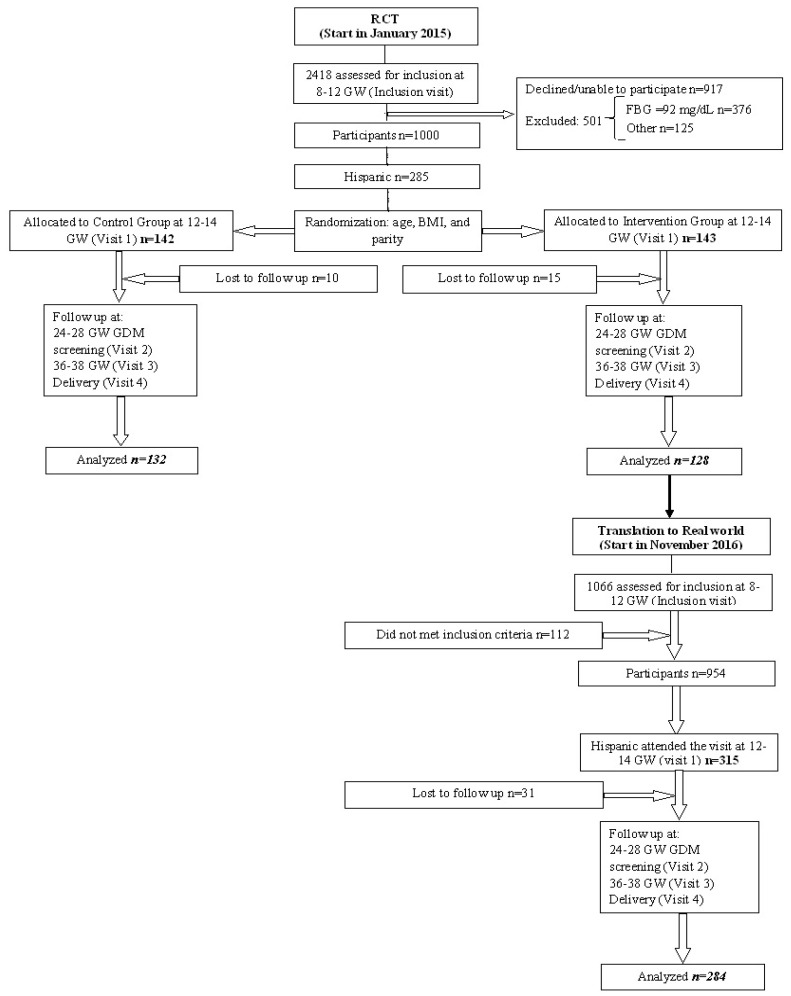
The flow chart shows the distribution of the participants. The diagram shows the flow of participants since the inclusion visit. The women who attended the first visit as well as those who completed the follow-up are shown in italics and bold.

The clinical flow chart is displayed in Figure 1.

#### 2.3.1. Clinical and Anthropometric Data

At visit 1 the years of residency in Spain, educational status, employment status, family history of type 2 diabetes mellitus and MetS, as well as prior gestational history (including miscarriages, GDM, and number of pregnancies) and gestational age (1st ultrasound) were collected. Information on any event related to their personal health, pharmacological treatments, and smoking habits (whether they were currently smoking, or they had continued smoking within the 6 months before knowing they were pregnant) was also gathered. In addition, pre-gestational body weight (BW) was self-referred, and height was measured in all women by an altimeter (Seca 799, seca GmbH & Co. KG, Hamburg, Germany).

Blood pressure (BP), BW, gestational weight gain (GWG), and BMI were evaluated and recorded at each visit. BP was assessed with a digital sphygmomanometer with adequate armlet after resting for 10 min in a sitting position (Omron 705IT, Omron Global, Kyoto, Japan). Further information can be found in the previous study [10,16].

#### 2.3.2. Biochemical Analysis

A blood sample was obtained after an overnight fast of 8–10 h at visits 1 to 3. The following data were determined: fasting serum glucose (glucose oxidase), and HbA1c (%) level, standardized by the International Federation of Clinical Chemistry and Laboratory Medicine, using ion-exchange high-performance liquid chromatography in gradient, with a Tosoh G8 analyzer (Tosoh Co., Tokyo, Japan). Inter-assay imprecision of HbA1c for levels of 5.1%: Standard Deviation (SD) of 0.06 and coefficient of variation (CV) of 1.23% and for levels of HbA1c 10.39%, SD is 0.11 and CV is 1.04. Fasting serum insulin (FSI), was measured by a chemiluminescence immunoassay in an IMMULITE 2000 Xpi (Siemens, Healthcare Diagnostics, Munich, Germany), with an inter-assay accuracy in concentrations of 11 uIU/mL of 6.3% and for insulin concentration of 21 uIU/mL of 5.91. Homeostasis assessment model for insulin resistance (HOMA) was calculated as glucose (mmol/L) × insulin (µUI/mL)/22.7. An External Quality Guarantee Program of the SEQC (Sociedad Española de Química Clínica) evaluates the quality of the methods monthly.

#### 2.3.3. Dietary and Lifestyle Assessment

Two semi-quantitative questionnaires of food frequency were filled out by a dietician during an interview with the participants at each visit to evaluate dietary intake, physical activity and adherence to nutritional therapy, as previously reported [10,16] (Table S2 online). In short, the lifestyle score consists of 15 items, 12 evaluating general eating habits and the last 3 physical activity, giving the Nutrition and Physical activity score. With this scoring system, Option A (value +1) is the most favorable result, whereas Option C (value −1) is the most unfavorable score. The nutrition score ranges from −12 to 12, with a desired goal above 5. The items pertaining to physical activity take into account daily walks, stair climbing, and at least 30 min of moderate intensity activities. The score ranged from −3 to 3. This questionnaire was previously validated in the Diabetes Nutrition and Complications Trial (DNCT), which was extensively detailed in the previous study [10]. The adapted form of the 14-point Mediterranean Diet Adherence Screener (MEDAS) was used to evaluate adherence to MedDiet. As previously reported, consumption of alcohol and juice were not considered because their intake is advised against during pregnancy. Thus, the MEDAS score ranged from 0 to 12 points. A detailed description can also be found in the prior study [10].

#### 2.3.4. Maternal, Delivery and Neonatal Outcomes

The Primary outcome was to assess the rate of GDM at 24–28 GW, as diagnosed by IADPSG criteria. Secondary maternal/delivery outcomes assessed were as follows: GWG, rates of pregnancy-induced hypertension (>140 mmHg systolic blood pressure (sBP) or >90 mmHg diastolic blood pressure (dBP) after 20 GW), preeclampsia (>140 mmHg systolic/90 mmHg diastolic with proteinuria >300 mg in 24 h after 20 GW, albuminuria (proteinuria >300 mg in 24-h with sBP <140 mm Hg and dBP <90 mm Hg), urinary tract infections (UTI) (as the number of events requiring antibiotic treatment), caesarean section (CS) and emergency CS, perineal trauma (as spontaneous tears without considering episiotomy), shoulder dystocia, and preterm delivery (<37 GW). Secondary neonatal outcomes assessed were: the number of newborns small for gestational age (SGA) (<10 percentile) and large for gestational age (LGA) (>90 percentile) according to national charts, neonatal hypoglycemia, hyperbilirubinemia, respiratory distress, and admissions to the Neonatal Intensive Care Unit (NICU), birthweight and length, Apgar test score, and the pH of cord blood.

### 2.4. Sample Size

For sample size calculation, the primary outcome was the incidence of GDM from 24 to 28 GW. The hypothesis of this study was that a nutritional therapy based on MedDiet early-on in pregnancy could be able to detect a relative risk reduction of at least 30% when comparing IG and RW with CG, allowing for a predicted drop-out rate of approximately 20% in accordance with the expected response rate. With 132 and 128 women assessed in RCT and 286 in RW, the study provided a statistical power of 80% (2-tailed, α error of 0.05) detecting a relative risk (RR) reduction of at least 30% in IG and RW vs. CG. 

### 2.5. Statistical Analysis

The categorical variables are expressed as number (%) and continuous variables are expressed as mean (SD). Comparison between groups (IG vs. CG and RW vs. CG) have been analyzed using the χ2 test for categorical variables and the Student’s t test or the Mann–Whitney U test for continuous variables as normal or not-normal distribution as verified by the Shapiro-Wilk test. Each group has been compared to baseline for trend using one-way analysis of variance (ANOVA). 

Logistic regression was used to assess the effect of the nutritional therapy for the maternal, delivery and neonatal adverse outcomes that were significantly different in the univariate analysis. Control Group was the reference Group. The relative risk (RR) and 95% confidence interval (95% CI) was adjusted by age, parity, and BMI.

All *p* values are 2-tailed at less than 0.05. Analyses were performed using SPSS, version 21 (SPSS, Chicago, IL, USA).

## 3. Results

A total of 132 women from IG, 128 from CG and 284 from RW completed all the visits and were evaluated at the end of the study. At baseline, no significant differences were observed in the semi-quantitative questionnaire scores of IG and RW when compared to the CG. Consumption of EVOO increased in all groups during pregnancy, but only remained significantly higher in the IG compared to CG. Pistachio consumption increased in IG and RW during pregnancy, with no modification in CG, although it was only significantly higher in IG and RW vs. GC during the 2nd trimester of gestation.

The Nutrition score increased throughout pregnancy in all three groups, being significantly higher in IG and RW compared to GC only during the 2nd trimester of gestation. Similarly, the MEDAS score was significantly increased in IG and RW, while no changes in the CG were observed, remaining significantly higher in RCT and RW groups compared to the CG throughout pregnancy. No significant differences in the level of physical activity were observed throughout pregnancy or between the groups. The data can be seen in Table 2.

A total of 34/132 women from CG were diagnosed with GDM between 24 and 28 GW, in contrast with the results obtained from IG and RW (19/128 and 38/284, respectively). The rate of GDM fell significantly from 25.8% of the CG to 14.8% of IG (*p* < 0.021) and 13.4% of the RW (*p* < 0.011) respectively. These data were associated with a significant decrease in HbA1c (%) values in the two latter groups at 24–28 GW and 36–38 GW compared with CG. The same decrease was observed in HOMA-IR, but only when comparing IG to CG. Furthermore, fewer IG and RW women diagnosed with GDM required insulin therapy when compared with CG: 12/34 (35.3%) CG; 4/19 [21.1% (*p* < 0.039)] IG, and 9/38 [23.7% (*p* < 0.001)] RW, respectively. The UTI rate was significantly lower in IG [7.0% (*p* < 0.003)] and RW [6.3% (*p* < 0.001)] vs. CG (18.9%). Similarly, the emergency CS rate was lower in the IG [1.6% (*p* < 0.020)] and RW [1.8% (*p* < 0.004)] compared to CG (7.6%). By the same token, the perineal trauma rate decreased from 11.4% in CG to 3.1% (*p* < 0.009)] in IG, and 1.6% (*p* < 0.001) in RW. A significant reduction in LGA and SGA rates were observed in IG compared to CG [0.8% vs. 6.1% (*p* < 0.02)] and [0.8% vs. 5.3% (*p* < 0.036)], respectively. These differences were not found in RW. These data are displayed in Table 3.

Logistic regression analysis of variables that were significant in the univariate analysis were adjusted for age (continuous), parity and BMI (continuous). The RR (95% CI) for GDM were [0.72 (0.50–0.97) (*p* = 0.037)] in the IG and [0.77 (0.61–0.97) (*p* = 0.008)], in the RW, respectively; the RR (95% CI) for other maternal complications can be found in Table 3. 

## 4. Discussion

In the current study, nutritional therapy based on MedDiet principles, with EVOO and pistachio supplements, decreased the incidence of GDM in Hispanic women. This was the case both in RCT, and when the diet was transposed onto routine management, in the RW group. Two recent reviews and meta-analyses indicate that early nutritional intervention is the most effective way to reduce the rate of GDM. The inclusion of foods with a low glycaemic index, as is the case of MedDiet with a high content in healthy fat, can also be effective [29,30]. Despite the fact that the rates of GDM in both IG and CG are high, after the medical nutrition therapy the difference between them is significant. When comparing GDM rate in the control group with rates found in the IG and RW groups, the decrease is over 40%.

Women diagnosed with GDM reached glycemic goals more frequently with nutritional treatment exclusively and required insulin treatment less frequently in the MedDiet intervention groups, RCT and RW. These data are similar to those previously described [28]. Likewise, the HbA1c was lower in the 3rd trimester in both MedDiet groups. However, the rates of HOMA-IR were lower in the IG, whereas they were similar in the RW vs. CG in the 2nd trimester and even higher in the 3rd. This can be explained by a decreased adherence to MedDiet in RW in the 3rd trimester, despite being greater than in the CG. While we observed a substantial improvement in dietary habits, it was still suboptimal, with less than 35% of RW women reaching the recommended EVOO intake. There was an improvement in the MEDAS and Nutrition scores during pregnancy in both IG and RW, achieving higher values in IG than in RW, and both remained higher than in CG. Despite this, there was also a decrease of the MEDAS scores in the 3rd trimester in the RW. Thus, maximum adherence in the RW is found within the 2nd trimester of gestation, and it may be sufficient to reduce the rate of occurrence of GDM. However, it does not seem to be enough to decrease the rate of LGA or SGA, which is only achieved in the IG. This is probably due to the lack of a supply of EVOO and pistachios at no cost in the RW group. Both products are expensive, and economic considerations can represent a barrier to their consumption [31,32]. Other factors must be considered. The social and cultural boundaries these women have to face seem to be related to adherence to healthy eating habits during pregnancy [33,34].

Significant results were also observed when analyzing maternal outcomes such as lower rates of emergency CS, UTI and perineal trauma in both IG and RW. These results could be explained by a possible relationship between MedDiet food components and their association with the immune system. The MedDiet is rich in phenolic compounds, which could be modulating the immune system and decreasing the rates of UTI. Furthermore, muscle energy deposit could improve labor, facilitating vaginal delivery and reducing delivery complications. [14,15]. Our data show a trend towards a decrease in emergency CS and perineal trauma. Recent evidence indicates that Hispanic women are at higher risk for emergency CS [35]. The Hispanic participants in our study show a lower rate of emergency CS than what has been described in studies from South American countries, where rates ranging from 32% in Peru to 34.8% in Ecuador have been found [36]. A study carried out in the United States found that Hispanic women who were immigrants presented a higher rate than those born in United States [37]. The Hispanic women participating in our study had a higher educational level and were more socially integrated than the women described in the United States study. Most of our Hispanic study subjects have been living in Spain for over 10 years. The acquisition of Spanish nutritional habits, with a high olive oil intake, possibly made the rates lower. Another significant difference from the United States is full access to medical care that is free at-point-of use in Spain, through the public National Health System, facilitating close medical supervision throughout pregnancy.

This study has several limitations. First, the sample included came from two consecutive studies. The fact that the two studies were not simultaneous could induce differences in the results found in the three groups. However, all participants in the RCT and RW groups received the same nutritional therapy, with the exception of free EVOO and pistachios in in the RCT group. Thus, lower rates in the primary outcome as well as in most of the secondary outcomes were observed in both groups. The second limitation is that the nutritional intervention was the same for all the Hispanic women, and the specific characteristics of the country of origin and its corresponding culture were not taken into account. However, participants had emigrated from several different Latin American countries. Thus, results could be extrapolated to women from Latin America in general. Another limitation is the fact that a food-frequency questionnaire could be biased by women’s answers and only offer an approximate picture of eating habits. However, semi-quantitative questionnaires offer a global view of food-frequency and evaluate multiple aspects of food intake in a quick and non-invasive fashion. For these reasons, these questionnaires are the tool of choice for a comprehensive view of adherence to diet in most nutrition studies.

## 5. Conclusions

Based on the results of the current study, a MedDiet-based nutritional intervention is associated with a decrease in the rates of GDM and several maternal-fetal adverse events in Hispanic women who are residents of Spain. Thus, this diet should be considered to be the nutritional therapy of choice in these women during pregnancy. Whether these conclusions are applicable for Hispanic women living in other countries remains to be elucidated. Public health strategies should be developed in Spain aimed towards increasing adherence to MedDiet in pregnant women.

## Figures and Tables

**Table 1:** Baseline Characteristics of Hispanic Women in the Randomized Clinical Trial (RCT) and the Real World (RW) Study.

	RCT		SS	RW Group	SS
	CG (*n* = 142)	IG (*n*= 143)	P	RW (*n* = 315)	*p*
Age (years)	31.3 ± 5.6	31.7 ± 5.4	0.573	31.4 ± 5.7	0.895
Years of Residency in Spain	10.0 ± 5.0	10.2 ± 5.8	0.773	10.1 ± 5.5	0.965
Family history of T2DM	37 (26.0)	41 (28.7)	0.102	90 (28.6)	0.520
MetS (>2 components)	20 (14.1)	20 (14.0)	0.402	46 (14.6)	0.101
Previous history of			0.449		0.127
- GDM	7 (4.9)	4 (2.8)		8 (2.5)	
- Miscarriages	57 (40.1)	62 (43.4)		160 (50.8)	
Educational status			0.067		0.127
Elementary School Education	28 (19.7)	18 (12.6)		35 (11.2)	
Secondary School Degree	67 (47.2)	66 (46.2)		160 (50.8)	
University Degree	45 (31.7)	58 (40.6)		112 (35.5)	
Unknown	2 (1.4)	1 (0.7)		8 (2.5)	
Unemployed	98 (69.0)	97 (67.8)	0.241	231 (73.3)	0.194
Number of pregnancies			0.172		0.660
Primiparous	36 (25.4)	42 (29.4)		97 (20.8)	
Second pregnancy	45 (31.7)	49 (34.3)		94 (29.8)	
>2 pregnancies	61 (42.9)	52 (36.3)		124 (39.4)	
Smoker			0.111		0.847
Never	105 (73.9)	96 (67.1)		241 (76.5)	
Current	1 (0.7)	1 (0.7)		5 (1.6)	
Gestational Age (weeks)	12.1 ± 0.8	12.0 ± 0.6	0.811	12.1 ± 0.6	0.791
Body Weight (kg)					
Pre-pregnancy	62.5 ± 12.2	60.2 ±9.6	0.068	58.9 ± 9.4	0.001
At baseline	64.3 ± 12.9	62.2 ± 9.7	0.061	60.9 ± 9.9	0.001
Weigth gain	2.3 ± 4.1	3.3 ± 1.6	0.188	2.1 ± 4.9	0.743
Pre-pregnancy BMI (kg/m^2^)	24.4 ± 4.0	24.1 ± 3.4	0.259	23.4 ± 3.6	0.033
Systolic BP(mm Hg)	105 ± 10	106 ± 10	0.443	108 ± 10	0.011
Diastolic BP (mm Hg)	63 ± 10	64 ± 9	0.684	66 ± 8	0.029
Fasting Blood Glucose (mg/dL)	81.3 ± 7.0	80.8 ± 6.0	0.604	80.2 ± 6.1	0.066
TSH mcUI/mL	2.0 ± 1.3	1.9 ± 1.5	0.575	1.9 ± 1.4	0.582
MEDAS Score	4.6 ± 1.8	4.2 ± 1.7	0.691	4.0 ± 1.6	0.323
Nutrition Score	−0.1 ± 3.3	−0.4 ± 3.1	0.510	−0.5 ± 3.0	0.423
Physical Activity Score	−1.6 ± 1.1	−1.8 ± 1.0	0.091	−1.8 ± 1.0	0.057

Data are Mean ± SD or number (%) RCT, Randomized Controlled Trial; CG, Control Group; IG, Intervention Group; RW, Real World group; MetS, Metabolic Syndrome; GDM, Gestational Diabetes Mellitus; BMI, Body Mass Index; BP, Blood Pressure; MEDAS Score, 14-point Mediterranean Diet Adherence Screener; Nutrition Score, a validated 12 food semi quantitative frequency questionnaire based on the Diabetes and Nutrition Complications Trial study, to assess the global lifestyle of the participants, considering a score of >5 the objective (range −12;12) Physical Activity Score, (Walking daily (>5 days ⁄ week): Score 0, at least 30 min; Score +1, if >60 min; Score −1, if <30 min. Climbing stairs (floors ⁄ day, >5 days a week): Score 0, between 4 and 16; Score +1, >16; Score −1: <4).

**Table 2:** Trends in lifestyle throughout pregnancy.

		AT BASELINE	24–28 GW	36–38 GW	P TREND
EVOO (mL/day)	CG	23 ± 22	28 ± 26	30 ± 22	0.020
	IG	29 ± 29	32 ± 19	40 ± 33	0.001
	p IG vs. CG	0.100	0.023	0.043	
	RW	23 ± 21	28 ± 27	28 ± 23	0.012
	p RW vs. CG	0.873	0.123	0.143	
Pistachio/Nuts (days/week)	CG	0.8 ± 1.6	1.0 ± 1.9	2.0 ± 2.9	0.128
	IG	0.8 ± 1.5	2.9 ± 2.7	3.0 ± 2.5	0.001
	p IG vs. CG	0.885	0.001	0.063	
	RW	1.0 ± 1.8	1.9 ± 2.2	2.7 ± 2.2	0.001
	p RW vs. CG	0.123	0.043	0.965	
Nutrition Score	CG	−0.1 ± 3.3	0.2 ± 3.5	3.1 ± 4.0	0.001
	IG	−0.4 ± 3.1	3.3 ± 3.0	4.0 ± 3.7	0.001
	p IG vs. CG	0.510	0.001	0.112	
	RW	−0.5 ± 3.0	1.3 ± 3.5	2.8 ± 4.0	0.001
	p RW vs. CG	0.423	0.001	0.516	
MedDiet Score	CG	4.6 ± 1.8	5.7 ± 1.8	5.0 ± 2.0	0.098
	IG	4.2 ± 1.7	7.4 ± 1.4	7.5 ± 1.6	0.010
	p IG vs. CG	0.691	0.001	0.034	
	RW	4.0 ± 1.6	6.3 ± 1.8	5.9 ± 22	0.001
	p RW vs. CG	0.323	0.001	0.043	
Physical Activity Score	CG	−1.6 ± 1.1	−1.7 ± 0.9	−1.8 ± 0.6	0.081
	IG	−1.8 ± 1.0	−1.9 ± 0.9	−1.7 ± 0.8	0.601
	p IG vs. CG	0.091	0.168	0.299	
	RW	−1.8 ± 1.0	−1.7 ± 0.9	−1.5 ± 0.9	0. 078
	p RW vs. CG	0.057	0.260	0.065	

Data are mean ± SD or *n* (%). IG, Intervention Group; CG, Control Group; RW, Real World group; EVOO, Extra Virgin Olive Oil; MEDAS Score, adapted 14-point Mediterranean Diet Adherence Screener (MEDAS); Nutrition Score, Diabetes Nutrition and Complications Trial (DNCT); Physical Activity Score, (Daily walks (>5 days ⁄ week): Score 0, at least 30 min; Score +1, if >60 min; Score −1, if <30 min. Stair Climbing (floors⁄day, >5 days a week): Score 0, between 4 and 16; Score +1, >16; Score −1: <4). p, denote differences between groups each time (*t*-test) and each group compared to baseline for trend (ANOVA).

**Table 3:** Maternal Pregnancy and Neonatal Outcomes.

	RCT		SS vs. CG		SS vs. CG
	CG (*n* = 132)	IG (*n* = 128)	*p*	RW (*n* = 284)	*p*
***MATERNAL OUTCOMES***					
GDM	34 (25.8)	19 (14.8)	0.021	38 (13.4)	0.011
RR (95% CI)	1	0.72 (0.50–0.97)	0.037	0.77 (0.61–0.97)	0.008
75 g-OGTT 24–28 GW					
Fasting Blood Glucose (mg/dL)	86.3 ± 7.0	84.3 ± 6.7	0.022	84.7 ± 6.2	0.018
≥92 mg/dL	24 (18.2)	17 (13.3)	0.167	28 (8.8)	0.032
RR (95% CI)	1	0.81 (0.55–1.19)	0.308	0.79 (0.61–1.03)	0.058
1 h Blood Glucose (mg/dL)	118.3 ± 34.7	116.5 ± 28.4	0.705	114.5 ± 27.3	0.569
≥180 mg/dL	6 (4.5)	2 (1.6)	0.146	4 (1.4)	0.077
RR (95% CI)	1	0.50 (0.15–1.66)	0.281	0.60 (0.28–1.28)	0.092
2 h Blood Glucose (mg/dL)	107.7 ± 24.3	104.5 ± 21.5	0.419	101.8 ± 21.4	0.054
≥153 mg/dL	9 (6.8)	4 (3.1)	0.130	5 (1.8)	0.016
RR (95% CI)	1	0.60 (0.27–1.38)	0.253	0.53 (0.26–1.00)	0.019
HbA1c (%) 24–28 GW	5.1 ± 0.3	5.0 ± 0.3	0.021	5.0 ± 0.3	0.001
>5.5%	12 (9.1)	5 (3.9)	0.081	6 (2.1)	0.004
HbA1c (%) 36–38 GW	5.5 ± 0.3	5.3 ± 0.2	0.001	5.3 ± 0.3	0.001
>5.5%	50 (38.0)	20 (15.2)	0.002	59 (20.8)	0.015
RR (95% CI)	1	0.49 (0.33–075)	0.003	0.44 (0.22–0.87)	0.024
FBG 36–38 GW (mg/dL)	78.4 ± 9.4	74.8 ± 4.4	0.006	79.5 ± 10.8	0.495
Fasting Serum Insulin (mcUI/mL)					
24–28 GW	9.9 ± 5.8	9.8 ± 4.5	0.787	9.7 ± 6.9	0.232
36–38 GW	12.4 ± 14.8	12.1 ± 11.6	0.846	15.7 ± 20.0	0.258
HOMA-IR					
24–28 GW	2.4 ± 1.4	2.0 ± 1.0	0.045	2.4 ± 3.8	0.835
36–38 GW	2.6 ± 4.3	2.2 ± 2.3	0.050	3.6 ± 5.8	0.225
Treatment of GDM					
Nutritional	22 (64.7)	15 (78.9)		29 (76.3)	
Insulin	12 (35.3)	4 (21.1)	0.039	9 (23.7)	0.001
Basal	10 (83.3)	2 (50)		5 (55.5)	
Basal/Bolus	2 (16.7)	2 (50)		4 (44.5)	
RR (95% CI) for IT	1	0.44 (0.12–1.00)	0.041	0.61 (0.38–0.96)	0.049
Weight gain (Kg) to 24–28 GW	7.6 ± 4.8	6.7 ± 3.8	0.102	6.9 ± 4.6	0.237
Weight gain (Kg) to 36–38 GW	11.3 ± 6.3	12.3 ± 5.4	0.209	12.5 ± 6.6	0.075
Systolic BP (mm Hg) 24–28 GW	104 ± 11	106 ± 11	0.131	105 ± 11	0.164
Diastolic BP (mm Hg) 24–28 GW	63 ± 9	63 ± 9	0.864	63 ± 9	0.413
Systolic BP (mm Hg) 36–38 GW	115 ± 16	113 ± 13	0.631	115 ± 14	0.256
Diastolic BP (mm Hg) 36–38 GW	72 ± 9	72 ± 9	0.192	71 ± 10	0.589
Pregnancy-induced					
hypertension	8 (6.1)	7 (5.5)	0.525	6 (2.1)	0.050
Preeclampsia	6 (4.5)	5 (3.9)	0.521	4 (1.4)	0.245
Albuminuria	3 (2.3)	0 (0)	0.129	0 (0)	0.037
Urinary Tract Infection	25 (18.9)	9 (7.0)	0.003	18 (6.3)	0.001
RR (95% CI)	1	0.53 (0.30–0.94)	0.008	0.60 (0.41–0.86)	0.001
Delivery					
Vaginal	95 (72.0)	92 (71.9)		192 (67.6)	
Instrumental	14 (10.6)	16 (12.5)		32 (11.3)	
Cesarean section (CS)	23 (17.4)	20 (15.6)	0.848	60 (21.1)	0.708
Emergency (CS)	10 (7.6)	2 (1.6)	0.020	5 (1.8)	0.004
RR (95% CI) for Emergency CS	1	0.70 (0.22–2.27)	0.383	0.15 (0.04–0.50)	0.001
Perineal Trauma	15 (11.4)	4 (3.1)	0.009	4 (1.6)	0.001
RR (95% CI)	1	0.52 (0.24–0.99)	0.033	0.31 (0.13–0.75)	0.001
***NEONATAL OUTCOMES***					
SHOULDER DYSTOCIA	1 (0.8)	0 (0)	0.508	1 (0.4)	0.315
Gestational Age at birth (weeks)	39.4 ± 1.4	39.3 ± 1.3	0.561	39.5 ± 1.5	0.359
<37 GW	8 (6.1)	4 (3.1)	0.203	15 (5.3)	0.337
<34 GW	0	0		1 (0.4)	
Birthweight (g)	3260 ± 437	3243 ± 363	0.518	3297 ± 471	0.789
Percentile	50.8 ± 25.9	52.7 ± 25.4	0.677	51.7 ± 28.8	0.882
Length (cm)	49.3 ± 2.2	49.1 ± 2.0	0.560	49.5 ± 2.1	0.516
Percentile	43.5 ± 28.7	41.1 ± 28.1	0.597	44.7 ± 29.2	0.874
LGA >90 percentile	8 (6.1)	1 (0.8)	0.020	11 (3.9)	0.457
>4500 g	2 (0.5)	0		3 (1.1)	
SGA <10 percentile	7 (5.3)	1 (0.8)	0.036	9 (3.2)	0.307
Ph Cord Blood	7.30 ± 0.27	7.29 ± 0.07	0.573	7.28 ± 0.08	0.202
≤7	0 (0)	0 (0)		2 (0.7)	0.435
Apgar Score at 1 min	8.8 ± 0.7	8.8 ± 0.7	0.830	8.73 ± 1.0	0.271
<5	3 (2.2)	3 (2.3)	0.967	3 (1.2)	0.088
Apgar Score at 5 min	9.9 ± 0.6	9.8 ± 0.5	0.338	9.8 ± 0.8	0.058
<7	0	0		2 (0.8)	
Neonatal					
Hypoglycemia	2 (1.5)	2 (1.6)	0.677	1 (0.4)	0.599
Respiratory distress	2 (1.5)	2 (1.6)	0.677	0 (0)	0.135
Hiperbilurrubinemia	15 (11.4)	13 (10.2)	0.455	10 (3.5)	0.491
NICU admittance	5 (3.8)	5 (3.9)	0.606	7 (2.5)	0.273

Data are mean ± SD or *n* (%). RCT, Randomized Controlled Trial; RW, Real World group; CG, Control Group; IG, Intervention Group; GDM, Gestational Diabetes Mellitus; IT, Insulin Treatment; BP, Blood Pressure; LGA, Large for Gestational Age; SGA, Small for Gestational Age; NICU, Neonatal Intensive Care Unit; RR (95% CI), Relative Risk (95% Confidence Interval).

## Data Availability

The main investigator had the overall responsibility to ensure that the participants’ anonymity is protected. The trial staff ensured that the participants’ anonymity was maintained. Metada are available as supplementary material. The datasets generated during the current study are available from the corresponding author on reasonable request.

## Supplementary Materials

The following are available online at https://www.mdpi.com/2072-6643/12/11/3505/s1, Table S1 online: Country of origin of Hispanic participants, Table S2 online: Nutrition and Physical Activity score derived from Diabetes Nutrition and Complications Trial (DNCT).
