# Supplementary material for: Effect of a Mediterranean Diet-Based Nutritional Intervention on the Risk of Developing Gestational Diabetes Mellitus and Other Maternal-Fetal Adverse Events in Hispanic Women Residents in Spain
Source: Nutrients. 2020 Nov 14;12(11):3505. doi: 10.3390/nu12113505 (PMC7696021; doi:10.3390/nu12113505)
Supplement: Supplementary file 1 [file nutrients-12-03505-s001.pdf]

Table 1. online. Country of origin of Hispanic participants.

	RCT			Real World Group	
	CG = 142	IG = 143	P	RW = 315	*p*
	**COUNTRY**				
**ARGENTINA**	0	3		8	
**BOLIVIA**	16	16		34	
**BRAZIL**	5	2		8	
**CHILE**	2	1		1	
**COLOMBIA**	11	14		25	
**CUBA**	1	2		3	
**ECUADOR**	39	39		70	
**HONDURAS**	6	8	0.054	21	0.093
**MEXICO**	1	1		1	
**NICARAGUA**	0	1		3	
**PARAGUAY**	22	7		45	
**PERU**	14	27		44	
**DOMINICAN REPUBLIC**	21	8		38	
**URUGUAY**	1	0		1	
**VENEZUELA**	3	7		13	

Abbreviations: RCT, Randomized Controlled Trial; CG, Control Group; IG, Intervention Group; RW, Real World group;

Table 2 online. Nutrition and Physical Activity score derived from Diabetes Nutrition and Complications Trial (DNCT)

	Score		
	**+1**	**0**	**-1**
**Physical Activity Score**			
1. Daily walks (≥ 5 days/week)	>1 h	At least 30 minutes	<30 minutes
2. Stair climbing (no floors/day, ≥ 5 day a week)	>16	4–16	<4
3. At least 30 minutes of more than moderate intensity	>3 day/week	2 or 3 day/week	<2 day/week
**Nutrition Score**			
**Servings per week**			
1. Vegetables	>12	6–12	<6
2. Fruits (pieces)	>12	6–12	<6
3. Nuts	>3	1–3	<1
4. Extra Virgin Olive oil	daily	≥3 day	<3 day
5. High-fat fish or Iberian ham	>3	1–3	<1
6. Bread and cereals (high fiber content)	>6	3–6	<3
7. Legumes	>2	1–2	<1
8. Low-fat milk and cheeses	>6	3–6	<3
9. Red meat and/or processed meat	<3	3–6	>6
10. Sauces (except homemade "sofritos")	<2	2–4	>4
11. Juices and sugar-sweetened beverages	<2	2–4	>4
12. Cookies	<2	2–4	>4

after A. Duran et al Journal of Diabetes 2 (2010) 203–211doi: 10.1111/j.1753-0407.2010.00081.x
